# Integrated meta-analysis, network pharmacology, and molecular docking to investigate the efficacy and potential pharmacological mechanism of Kai-Xin-San on Alzheimer's disease

**DOI:** 10.1080/13880209.2020.1817103

**Published:** 2020-09-21

**Authors:** Pengji Yi, Zheyu Zhang, Siqi Huang, Jiahua Huang, Weijun Peng, Jingjing Yang

**Affiliations:** a Department of Integrated Traditional Chinese and Western Medicine, the Second Xiangya Hospital, Central South University, Changsha, China; b Department of Gastroenterology, Xiangya Hospital, Central South University, Changsha, China; c Hunan Academy of Chinese Medicine, Changsha, China; d Teaching and Research Section of Clinical Nursing, Xiangya Hospital, Central South University, Changsha, China; e Xiangya Nursing School, Central South University, Changsha, China

**Keywords:** Traditional Chinese medicine formula, dementia, pharmacological network, molecular docking technology

## Abstract

**Context:**

Kai-Xin-San (KXS) has been used to treat Alzheimer’s disease (AD) for thousands of years. However, no quantitative data regarding AD treatment using KXS are available. Moreover, its active compounds and mechanism of action for the treatment of AD remain largely unclear.

**Objectives:**

To evaluate the efficacy and the potential pharmacological mechanisms of KXS in AD treatment.

**Materials and methods:**

A systematic collection of KXS experiments was conducted from PubMed, Web of Science, Embase, CNKI, VIP, and Wanfang Data up to February, 2020. Review Manager 5 software was used for meta-analysis. In network pharmacology, components of KXS were screened, AD-related genes were then identified and the ‘component-target-pathway’ network constructed. Molecular docking was finally employed for *in silico* simulation matching between representative KXS compounds and their target genes.

**Results:**

Meta-analysis revealed that KXS improves the cognitive benefits in AD models by reducing the time of escape latency (SMD = −16.84) as well as increasing the number of cross-platform (SMD = 2.56) and proportion of time in the target quadrant (SMD = 7.52). Network pharmacology identified 25 KXS active compounds and 44 genes targets. DRD2, MAOA, ACHE, ADRA2A and CHRM2 were core target proteins. Besides, 22 potential pathways of KXS were identified, like cholinergic synapses, the cGMP/PKG pathway and calcium signalling. Molecular docking showed that stigmasterol, aposcopolamine and inermin can closely bind three targets (ACHE, ADRA2A and CHRM2).

**Discussion and conclusion:**

These findings suggest that KXS exerts effect on AD through multi-target, multi-component and multi-pathway mechanism. Future studies may explore the active components of KXS.

## Introduction

Alzheimer's disease (AD) is the most common cause of dementia. It is a progressive and unremitting neurodegenerative disease characterised by memory loss and cognitive decline (Fang et al. 2017). Alzheimer’s Association (AA) 2019 report projects that by 2050, more than 100 million individuals worldwide will suffer from Alzheimer’s dementia. AD causes great suffering to patients and their families as well as imposing a huge burden on society (AA 2016). Despite this, there are currently no effective treatments that can inhibit the progression of AD (AA 2019). As such, developing a new effective therapy for the treatment of AD is of utmost importance.

Kai-Xin-San (KXS) is a famous traditional Chinese medicine formula that has been proven to exert a certain effect on AD. The formula was first recorded in the book titled *‘Thousand Emergency Formulas’* authored by Simiao Sun in the Tang Dynasty (Cao et al. 2018). The formula is generated using four herbs: Ginseng Radix et Rhizome (*Panax ginseng* C. A. Mey. [Araliaceae], Renshen), Polygalae Radix (*Polygala tenuifolia* Wild. [Polygalaceae], Yuanzhi), Poria (*Poria cocos* (Schw.) Wolf [Polyporaceae], Fuling), and Acori Tatarinowii Rhizoma (*Acorus tatarinowii* Schott [Araceae], Shichangpu) in the ratio of 1:1:50:25. Wang et al. (2019) reported that KXS alleviated cognitive deficits of AD transgenic mice by repairing Aβ1-42 deposition damage. In the same line, Dang et al. (2009) reported that KXS could also modulate neurotransmitters such as cholinergic and monoamine to show nootropic effects. Similarly, Cao et al. (2018) reported that KXS could increase the expressions of neurotrophic factor NGF and BDNF in mouse astrocyte cultures. This was an indication that it has neuroprotective effects.

Despite KXS’s long history of use and abundant clinical support, its efficacy in the treatment of AD has not been fully validated. Additionally, its active ingredients and their underlying mechanism of action have not been fully elucidated.

In the recent past, meta-analysis of animal experiments have enabled rational evaluation of research evidence thus opening up avenues to evaluate KXS’s promising experimental interventions in clinical trials (Wang et al. 2015). Network pharmacology explains the pharmacological mechanism of drug formulas in a holistic manner. It helps in improving their clinical efficacy as well as reduce their toxicity and side effects in a time and labour efficient manner (Hopkins 2008; Li and Zhang 2008).

Herein, meta-analysis studies were done to evaluate the efficacy of KXS in the treatment of AD. Further to this, the network pharmacology approach was used to explore the potential pharmacological mechanisms of KXS in the treatment of AD. Molecular docking was further conducted to determine the binding efficiency of KXS compound-putative target pairs.

## Materials and methods

### Meta-analysis to evaluate the treatment effect of KXS on AD

#### Systematic search

A systematic search was conducted in six electronic databases to understand AD treatment using KXS. The six databases were: PubMed, Web of Science, Embase, China National Knowledge Infrastructure (CNKI), the VIP information resource integration service platform (VIP), and Wanfang Data knowledge service platform (Wanfang Data). The dates ranged from the establishment to February 2020. The search terms used were: Alzheimer’s disease, Alzheimer disease, dementia, KaiXinSan, and Kai-Xin-San.

#### Inclusion criteria

Studies involving animal models of dementia whose intervention was KXS therapy were included in the meta-analysis. Studies whose behaviour testing outcomes were used to evaluate cognitive functions were also included in the analyses.

##### Exclusion criteria

Duplicate articles, reviews, meeting abstracts and those that had no animal experiments were excluded from the study. Studies involving other traditional Chinese herbs were also excluded.

#### Data extraction

Data regarding article information (author and publication year), animal species, gender of the animals, the type of models, dosage of KXS used and the modalities of outcome assessment were collected from each study. Only the high-dosage data were collected in studies where different KXS dosages were used. Similarly, only the final time-point data were extracted in studies where behavioural tests were performed. Data expressed graphically were extracted using GetData software.

#### Statistical analysis

The Stata software (Review Manager 5.3) was used for meta-analysis. Based on the Cochrane Handbook for Systematic Reviews, outcome data such as escape latency, time in the target quadrant and the number of platform crossings data was considered as continuous data. The standard mean difference (SMD) and 95% confidence interval (CI) was determined using Review Manager 5.3. A random-effects model was also performed on the data to estimate the combined effect sizes. Q statistic and I^2^ statistics were used to quantifying heterogeneity.

### Network pharmacology-based predicting of the potential actions of KXS on AD

#### Data preparation

Data preparation to collect network pharmacology was done using several databases. The databases used were: The Traditional Chinese medicine system pharmacology technology platform (TCMSP, http://tcmspw.com/tcmsp.php), The Encyclopaedia of Traditional Chinese Medicine (ETCM, http://www.nrc.ac.cn:9090/ETCM/index.php/Home/Index/index.html), GeneCards (https://www.genecards.org/), Online Mendelian Inheritance in Man (OMIM, http://www.omim.org/), STRING (http://stringdb.org/cgi/input.pl), Pubchem (https://pubchem.ncbi.nlm.nih.gov/) and Protein Data Bank (PDB, http://www.rcsb.org/).

#### Building of an ingredient database

KXS compounds were obtained from the TCMSP and ETCM databases. They were then fed into the TCMSP database to determine their active ingredients. The TCMSP database provide herbal information from the Chinese Pharmacopoeia, while TTD and PharmGKB provide disease information. The TCMSP database also provides information on medicinal chemistry, drug similarities, drug targets, diseases targeted by each active compound as well as other associated information (Ru et al. 2014).

#### Prediction of drug-likeness, oral bioavailability, and blood-brain barrier

Biological experiments require a considerable amount of time and human resources. Cognisant to this, ADME (absorption, distribution, metabolism, and excretion) studies are used to assess the drug transport process because it provides a convenient approach for research (Xie et al. 2018). Herein, oral bioavailability (OB), blood-brain barrier (BBB), and drug-likeness (DL) were used to screen for the active components of the drug (Pang et al. 2018; Xie et al. 2018).

OB is one of the most important pharmacokinetic parameters. It represents the rate and extent of active ingredient absorption (Xu et al. 2012). BBB is postulated to be strongly related to the dysfunction and degradation of neural networks. As such, it must be circumvented to allow drugs to enter the brain (Friedman and Kaufer 2015). DL is used to filter out poorly performing compounds based on their respective molecular structures or structural units in drugs or drug-like molecules. This further optimises pharmacokinetics (Tian et al. 2015).

In this study, compounds with OB ≥ 30%, BBB ≥ −0.3, and DL ≥ 0.18 were selected for subsequent analysis.

#### Prediction of targets

AD targets obtained from the GeneCards and OMIM databases were used for further evaluation. GeneCards is an integrative database with information on human genes, diseases, proteins and cells. It is used to effectively navigate the links between them (Stelzer et al. 2016). The OMIM database is a comprehensive repository of genes and genetic phenotypes (Amberger and Hamosh 2017).

#### Protein–protein interaction (PPI) and enrichment analysis

The obtained targets were submitted to the STRING database for protein–protein interaction (PPI) analysis. The species was limited to *Homo sapiens* and confidence scores limited to those >0.4 (Szklarczyk et al. 2015). In the same line, the targets were subjected to Gene Ontology (GO) and Kyoto Gene and Genomic Encyclopaedia (KEGG) pathway for enrichment analysis using the R software (Version 3.26).

#### Construction of related networks

Three networks were constructed: (1) active compound-target network in AD treatment, (2) PPI network and (3) active compound-target-pathway in AD treatment.

Cytoscape 3.7.1 integrates molecules’ molecular interaction networks into a unified framework. Through its plug-ins, the framework can be extended to perform analytical calculations and provide visualisations for complex network analysis (Shannon et al. 2003).

#### Molecular docking

Molecular docking was performed to predict the interaction of the targets with the compounds. The crystal structure of the protein was downloaded from the PDB library and introduced into the Molecular Operating Environment software (Version 2015.10) for protein structure construction. It was subsequently modified by removal of water, protonation and energy minimisation to construct the protein structure. Similarly, compounds downloaded from the PubChem database were introduced into the ChemBioDraw software to produce their three-dimensional structures. The protein structure constructed using the Molecular Operating Environment software matched the three-dimensional structure of the compound.

## Results

### Meta-analysis

**Figure 1.**
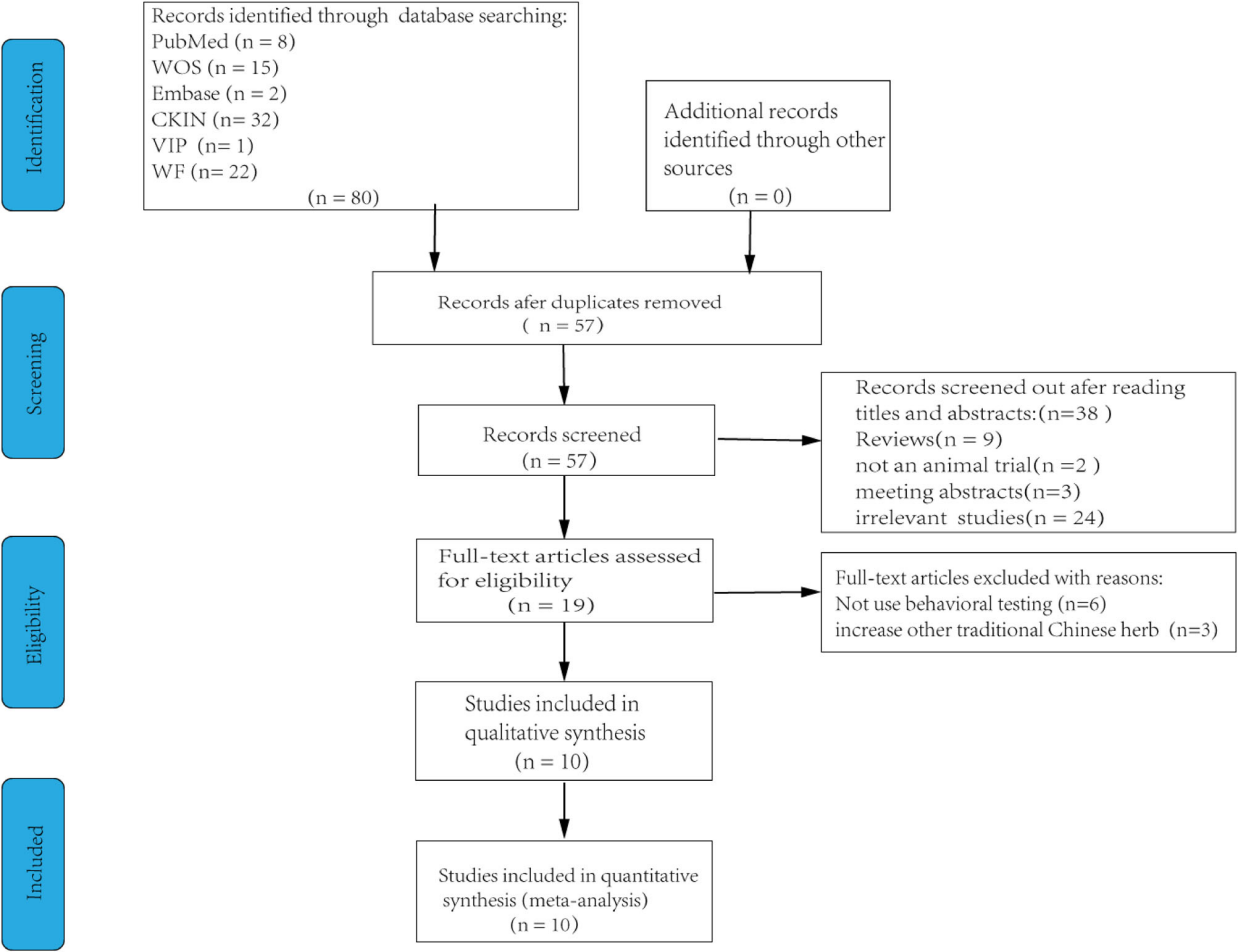
Flow diagram of the search process.

**Figure 2.**
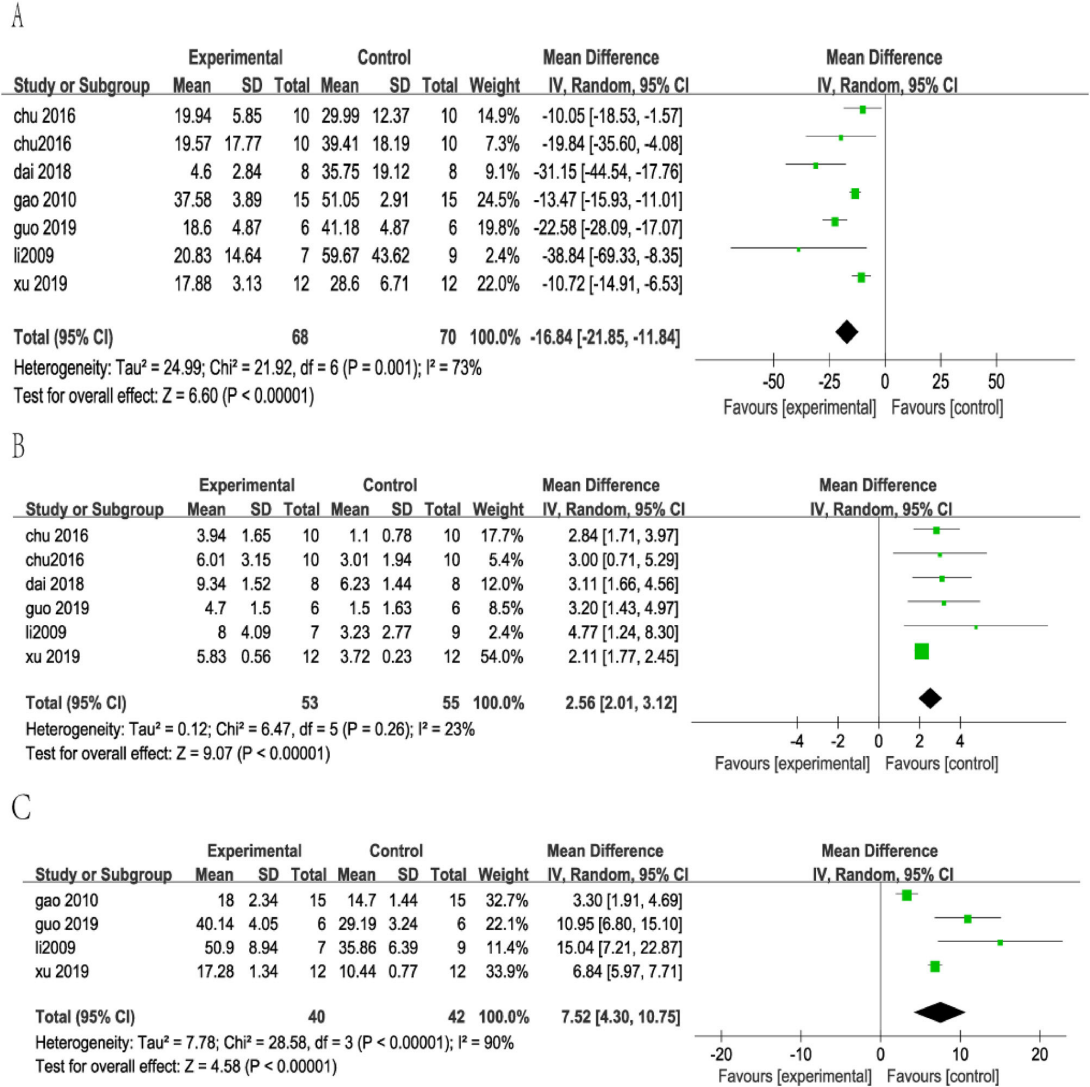
Forest plot showed the effect of KXS therapies on outcomes of the water maze.

Ten studies (Huang et al. 1999; Li and Zhao 2009; Gao et al. 2010; Chu et al. 2016a, 2016b; Lu et al. 2017; Dai et al. 2018; Guo et al. 2019; Xu et al. 2019; Zhang et al. 2019) were included in statistical analysis after screening 80 studies (Figure 1). The escape latency outcome was measured in seven of these studies (Li and Zhao 2009; Gao et al. 2010; Chu et al. 2016a, 2016b; Dai et al. 2018; Guo et al. 2019; Xu et al. 2019). KXS treatment was found to significantly reduce escape latency in AD animal models (SMD = −16.84, 95% CI = −21.85 to −11.84, *p* < 0.00001). The heterogeneity among studies was moderate (*I*^2^ = 73%, *p* = 0.001) (Figure 2(A)). Six studies (Li and Zhao 2009; Chu et al. 2016a, 2016b; Dai et al. 2018; Guo et al. 2019; Xu et al. 2019) evaluated the number of platform crossings for retention memory. In the same line, four studies (Li and Zhao 2009; Gao et al. 2010; Guo et al. 2019; Xu et al. 2019) adopted time in target quadrant as an outcome. The analysis revealed that AD model animals treated with KXS had a significant increase in the number of platform crossing compared with animals in the control groups (SMD = 2.56, 95% CI = 2.01–3.12, *p* < 0.00001). There was slight heterogeneity among studies (*I*^2^ = 23%, *p* = 0.26) (Figure 2(B)). Similarly, pooled analysis revealed that AD model animals treated with KXS had a significant increase in the proportion time compared with animals in the control groups (SMD = 7.52, 95% CI = 4.30–10.75, *p* < 0.00001). The heterogeneity among studies was large (*I*^2^ = 90%, *p* = 0.00001) (Figure 2(C)).

### Analysis of the properties of KXS

Based on the ADME index (OB ≥ 30%, BBB ≥ −0.3, DL ≥ 0.18), KXS was found to be comprised of 31 compounds (Table 1). The 31 compounds included two species of Acori Tatarinowii Rhizoma, six species of Poria, 21 species of Ginseng Radix et Rhizoma and two species of Polygalae Radix. Additionally, 53 predicted targets of KXS were found based on the obtained compounds. Removal of the target-free ingredients yielded 25 active compounds generated from the TCMSP database.

**Table 1.** 31 compounds of KXS.

MOL ID	Molecule name	OB (%)	BBB	DL
MOL003576	(1*R*,3a*S*,4*R*,6a*S*)-1,4-*bis*(3,4-Dimethoxyphenyl)-1,3,3a,4,6,6a-hexahydrofuro[4,3-c]furan	52.35	0.05	0.62
MOL003578	Cycloartenol	38.69	1.33	0.78
MOL000275	Trametenolic acid	38.71	−0.14	0.8
MOL000282	Ergosta-7,22E-dien-3beta-ol	43.51	0.91	0.72
MOL000283	Ergosterol peroxide	40.36	0.34	0.81
MOL000287	3Beta-Hydroxy-24-methylene-8-lanostene-21-oic acid	38.7	−0.04	0.81
MOL000296	Hederagenin	36.91	0.96	0.75
MOL000300	Dehydroeburicoic acid	44.17	−0.16	0.83
MOL002879	Diop	43.59	0.26	0.39
MOL000449	Stigmasterol	43.83	1	0.76
MOL000359	Beta-sitosterol	36.91	0.99	0.75
MOL003648	Inermin	65.83	0.36	0.54
MOL005308	Aposiopolamine	66.65	0.4	0.22
MOL005314	Celabenzine	101.88	0.05	0.49
MOL005317	Deoxyharringtonine	39.27	−0.25	0.81
MOL005320	Arachidonate	45.57	0.58	0.2
MOL005321	Frutinone A	65.9	0.46	0.34
MOL005348	Ginsenoside-Rh4_qt	31.11	−0.18	0.78
MOL005356	Girinimbin	61.22	1.22	0.31
MOL005357	Gomisin B	31.99	0.18	0.83
MOL005360	Malkangunin	57.71	−0.17	0.63
MOL005376	Panaxadiol	33.09	0.23	0.79
MOL005384	Suchilactone	57.52	0.28	0.56
MOL005399	Alexandrin_qt	36.91	0.88	0.75
MOL005401	Ginsenoside Rg5_qt	39.56	0.21	0.79
MOL000787	Fumarine	59.26	−0.13	0.83
MOL012254	Campesterol	37.58	0.98	0.71
MOL011093	Aposcopolamine	59.68	0.68	0.25
MOL008968	Gomisin A	30.69	−0.02	0.78
MOL002140	Perlolyrine	65.95	0.15	0.27
MOL003370	Onjixanthone I	79.16	0.04	0.3

### Prediction of AD-related targets

**Figure 3.**
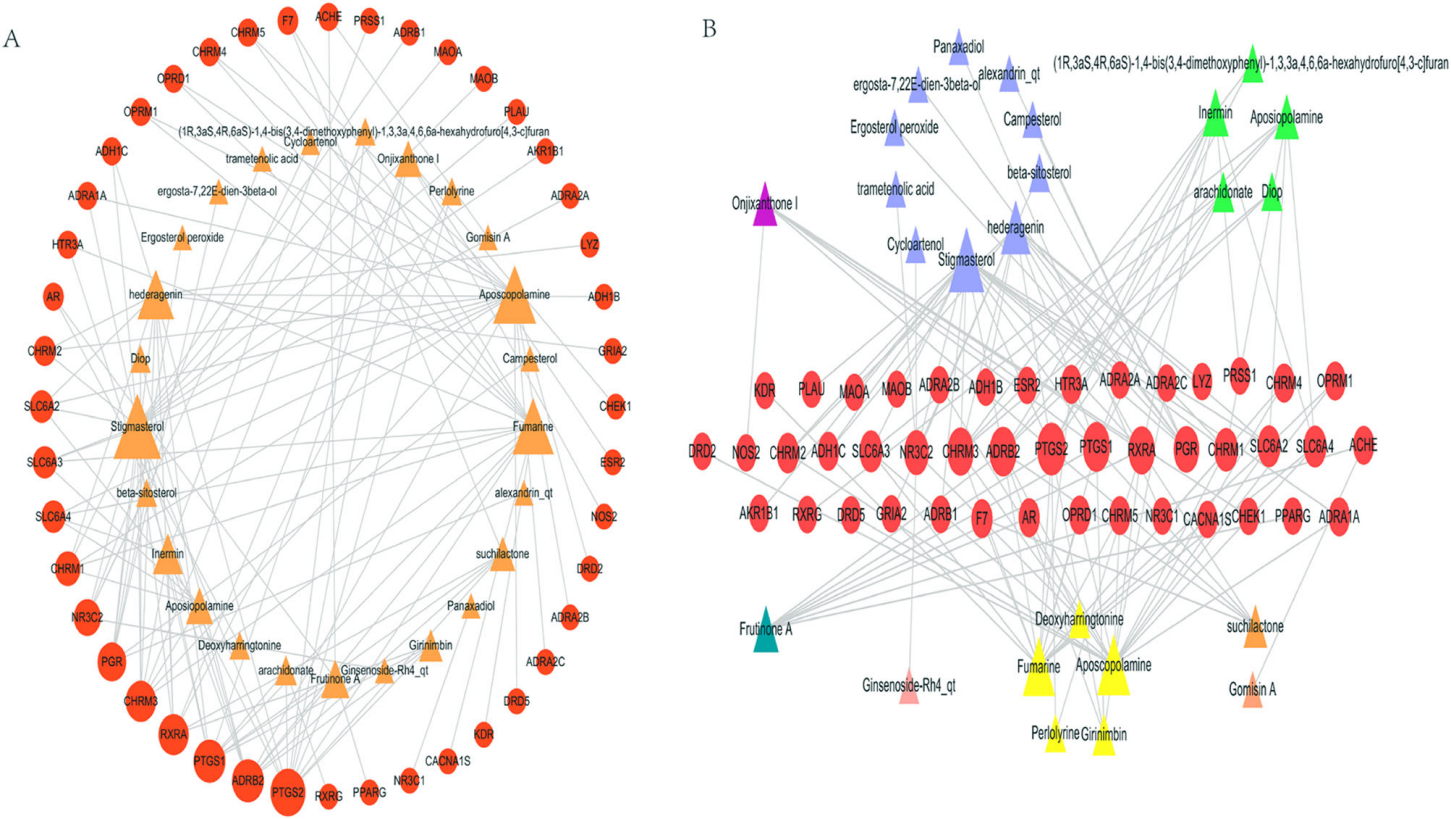
Compound-target of the treating AD network.

By removing duplicates, 7945 AD-related targets were obtained in the OMIM and GeneCard databases. Forty-four possible AD targets were subsequently obtained by matching the predicted KXS targets. The compound–target network was composed of 69 nodes (44 target nodes and 25 compound nodes) and 120 edges (Figure 3(A)). In this network, some targets such as PTGS2, PTGS1, ADRB2, CHRM3 and RXRA corresponded to multiple compounds. Similarly, several compounds such as stigmasterol, aposcopolamine, and fumarine corresponded to multiple targets. In the same line, several targets such as ESR2 and NOS2 were modulated by only one compound. These results suggested that KXS compounds acted synergistically on their targets based on the multi-compound and multi-target features of the herbal formula.

Based on the structural classification of traditional Chinese medicine compounds, the 25 active ingredients comprised of 10 terpenoids, 5 alkaloids, 2 lignin, 1 coumarin, 1 ketone, 1 saponin, and 5 types of other compounds. Terpenoids and alkaloids were found to be more closely associated with AD compared to the other compounds (Figure 3(B)).

### PPIs

**Figure 4.**
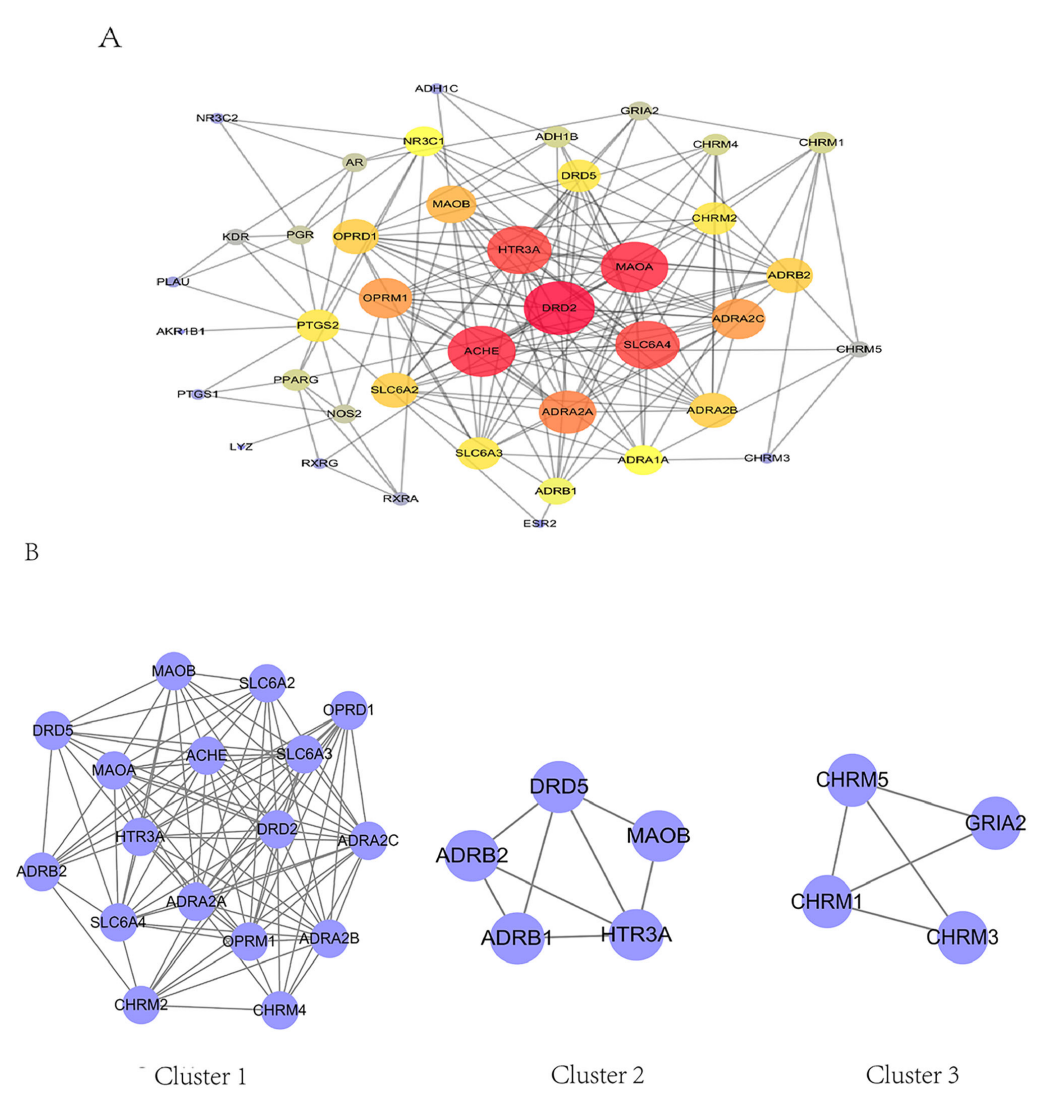
Protein–protein interaction network of treating AD.

Based on the results obtained from the STRING database, the relevant target protein was found to have 40 targets (4 target proteins were removed because they did not interact with other proteins). The PPI network contained 40 nodes and 177 edges (Figure 4(A)). In this network, the red nodes (DRD2, ACHE, MAOA, HTR3A, SLC6A4 and ADRA2A) had higher degree. Moreover, they had a large number of edge sides (19 for DRD2, 18 for ACHE, 18 for MAOA, 17 for HTR3A, 17 for SLC6A4 and 15 for ADRA2A). These results suggested that these genes play a critical role in development of AD.

Three functional clusters were constructed from the PPI via Molecular Complex Detection clustering (Figure 4(B), Table 2).

**Table 2.** Cluster of KXS PPI network.

Score	Nodes	Edges	Node IDs
10.167	13	61	SLC6A4, MAOA, CHRM2, OPRD1, OPRM1, ACHE, CHRM4, SLC6A2, SLC6A3, DRD2, ADRA2C, ADRA2A, ADRA2B
4	5	8	ADRB2, HTR3A, DRD5, MAOB, ADRB1
3.333	4	5	CHRM1, CHRM3, GRIA2, CHRM5

### GO, KEGG enrichment and compound–target–pathway networks of AD treatment

**Figure 5.**
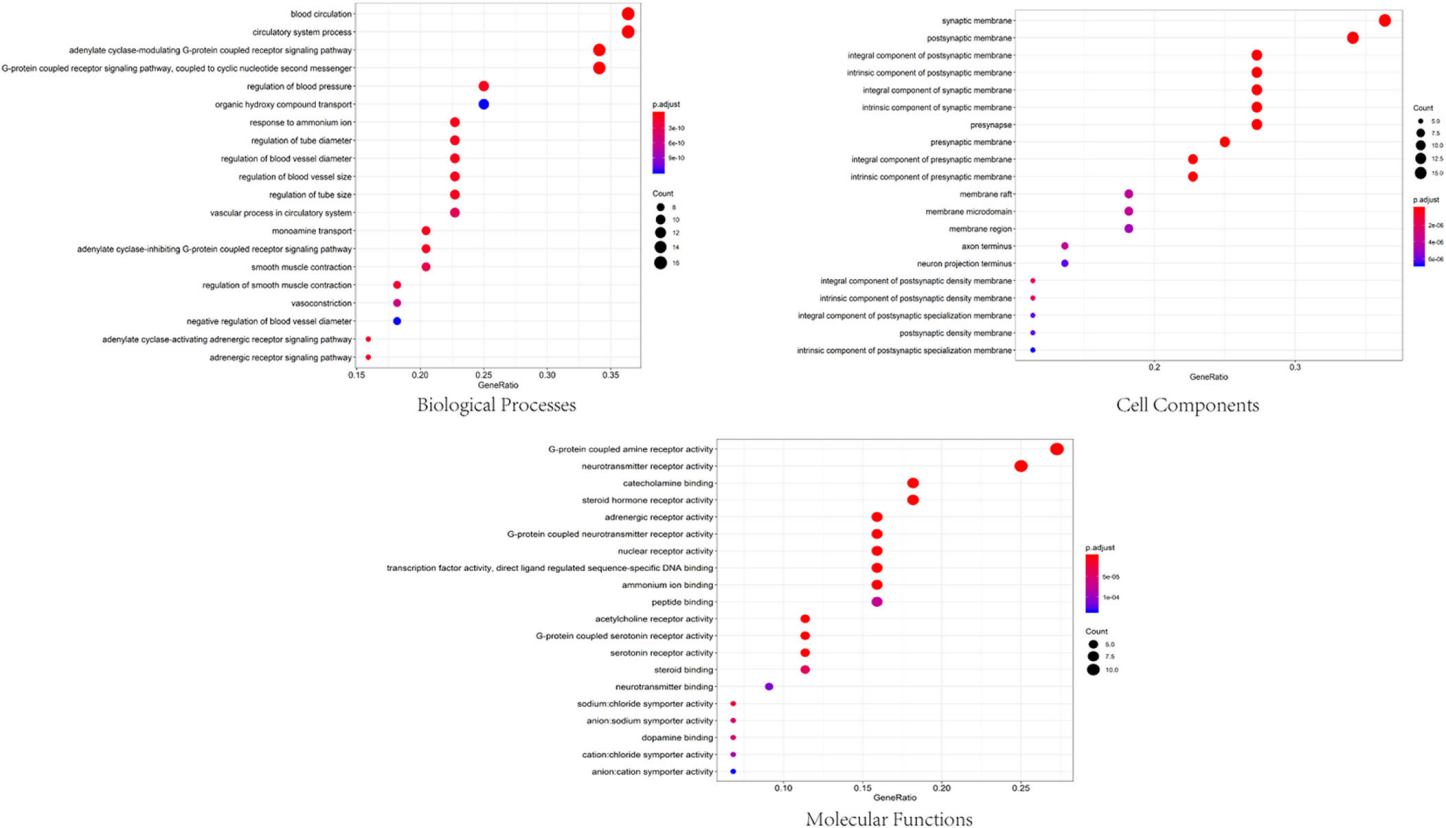
GO enrichment analysis of treating AD targets.

**Figure 6.**
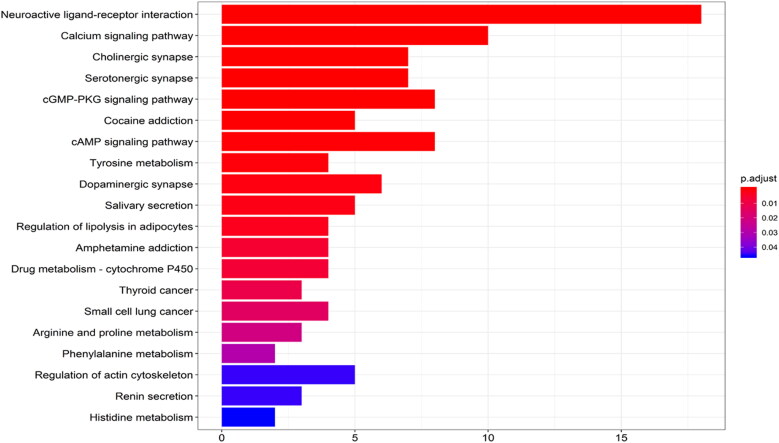
KEGG enrichment analysis of treating AD targets.

Forty-four target genes were significantly enriched in 389 biological processes, 72 molecular functions and 48 cell components (*p*-value <0.05). The top five terms in the biological processes were: adenylate cyclase-modulating G-protein coupled receptor signalling (GO: 0007188), G-protein-coupled receptor signalling (GO: 0007187), blood circulation (GO: 0008015), circulatory system (GO: 0003013), and regulation of blood pressure (GO: 0008217) (Figure 5). Regarding their molecular functions, the target genes were mainly enriched in acetylcholine receptor (GO: 0015464), catecholamine binding (GO: 1901338), neurotransmitter receptor (GO: 0030594), adrenergic receptor (GO: 0004935), and steroid hormone receptor (GO: 0003707). The postsynaptic membrane, presynaptic membrane, axon and neuron projection were the most common cell components associated with the target genes. KEGG analysis revealed that the compounds were mainly enriched in 22 signalling pathways (*p*-value <0.05) (Figure 6). The signalling pathways included neuroactive ligand–receptor interactions (hsa04080), calcium signalling pathways (hsa04020), cholinergic synapses (hsa04725), serotoninergic synapses (hsa04726), and cyclic guanosine monophosphate/protein kinase (GGMP/PKG) signalling pathways (hsa04022). These enrichment analyses suggested that KXS play a neuroprotective role by targeting the neurotransmission pathways.

**Figure 7.**
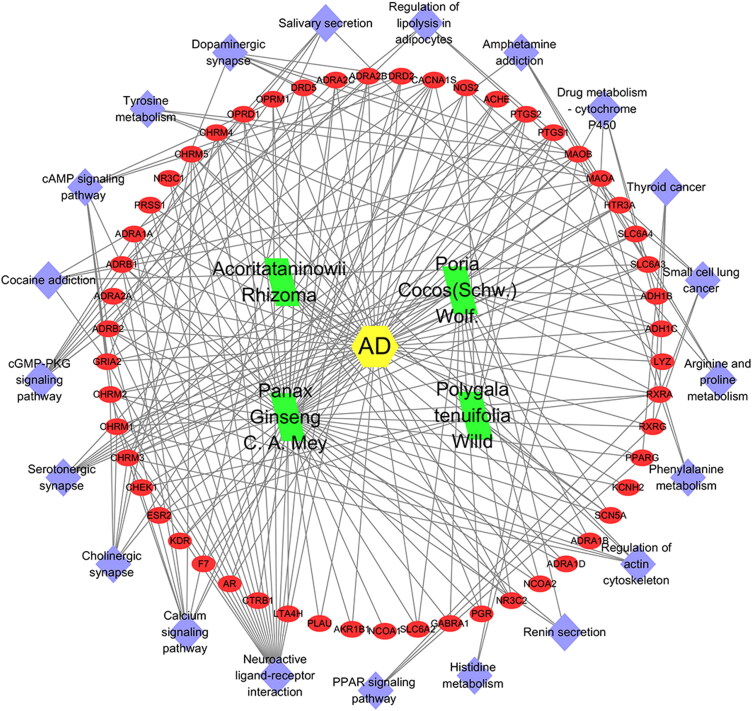
Compound–target–pathway of treating AD network.

A compound–target–pathway network associated with AD was constructed (Figure 7). The network revealed that KXS is associated with the treatment of AD through multiple targets and multiple pathways.

### Molecular docking analysis

ACHE (PDB code: 6O69), ADRA2A (PDB code: 5XZH), and CHRM2 (PDB code: 5ZK8) were selected as successful preparations of the receptor protein, followed by molecular docking with stigmasterol, aposcopolamine, and inermin.

**Figure 8.**
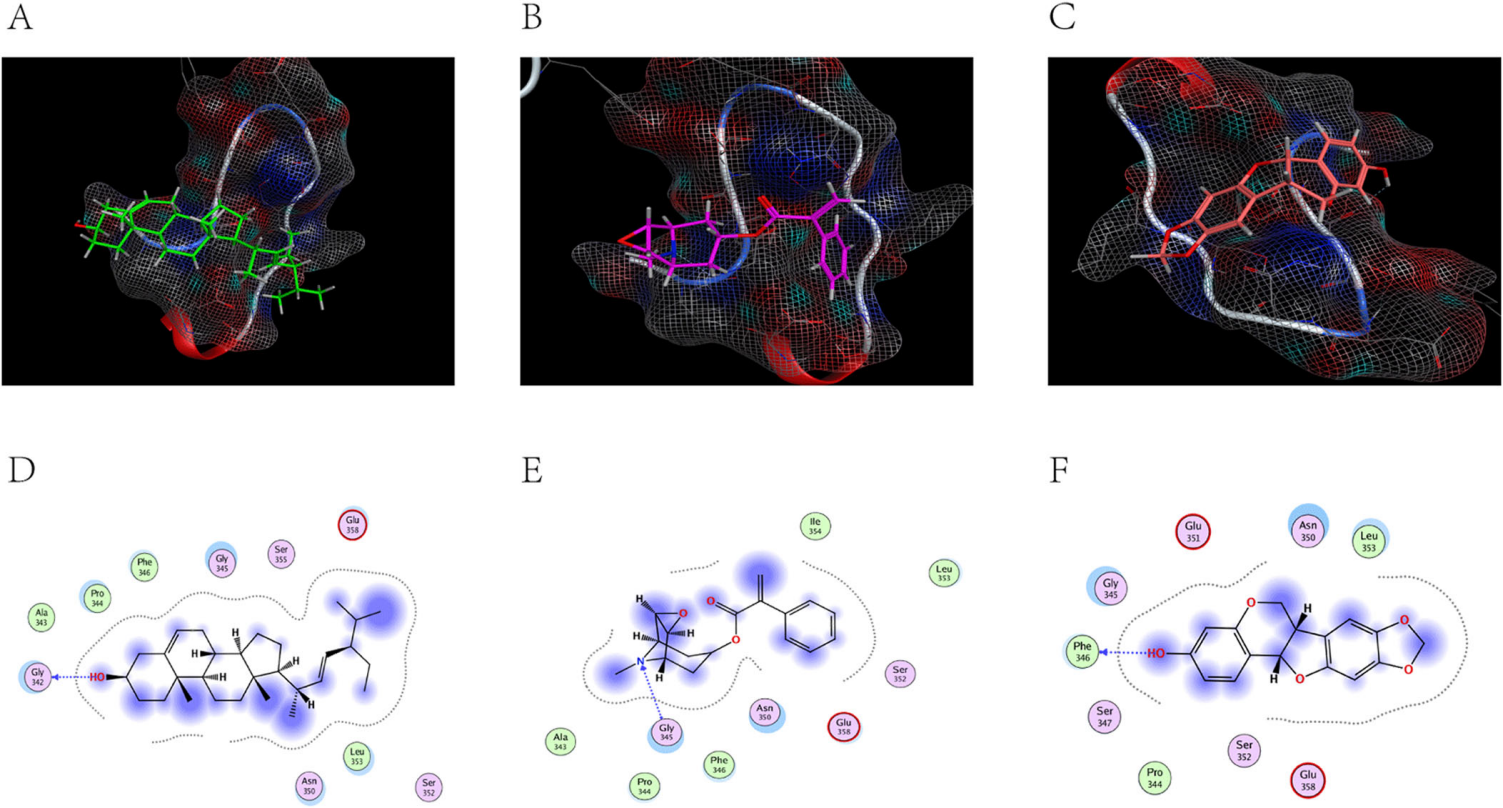
The docking model of compounds with ACHE.

Figure 8(A–C) are schematic views of ACHE linked to stigmasterol, aposcopolamine, and inermin, respectively. ACHE and stigmasterol formed hydrogen bonds at GLY 342 (Figure 8(D)). Figure 8(E) demonstrates that GLY 345 of aposcopolamine was joined to the N atom of ACHE by hydrogen bonding. In the same line, PHE 346 formed a hydrogen bond with ACHE in inermin (Figure 8(F)).

**Figure 9.**
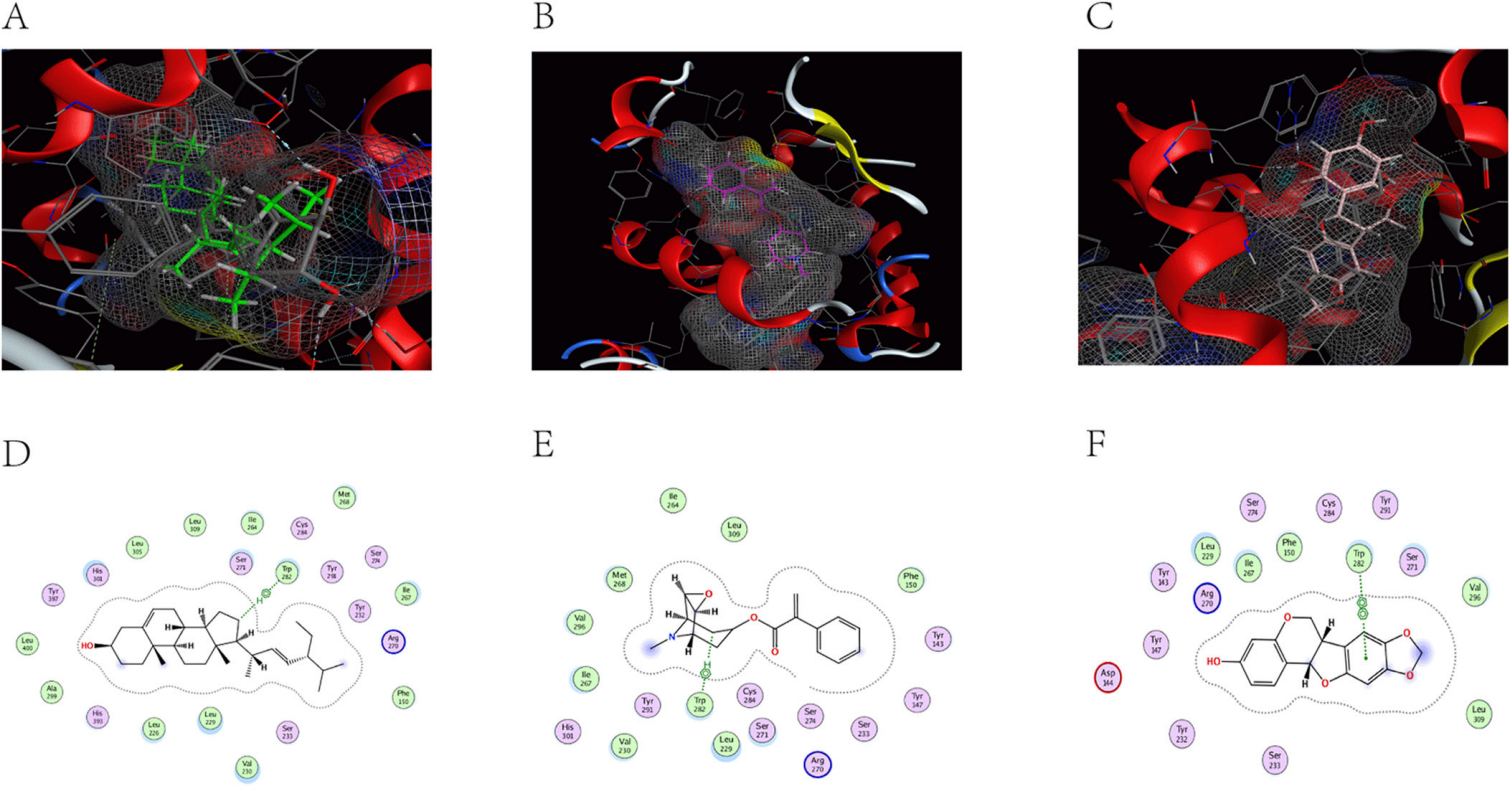
The docking model of compounds with ADRA2A.

ADRA2A and stigmasterol models are shown in Figure 9(A). TRP 282 connected to ADRA2A via π-H bonds. Similarly, aposcopolamine was also attached to ADRA2A via π-H bonds (Figure 9(E)), while inermin and ADRA2A were connected via π-π bonds (Figure 9(F)).

**Figure 10.**
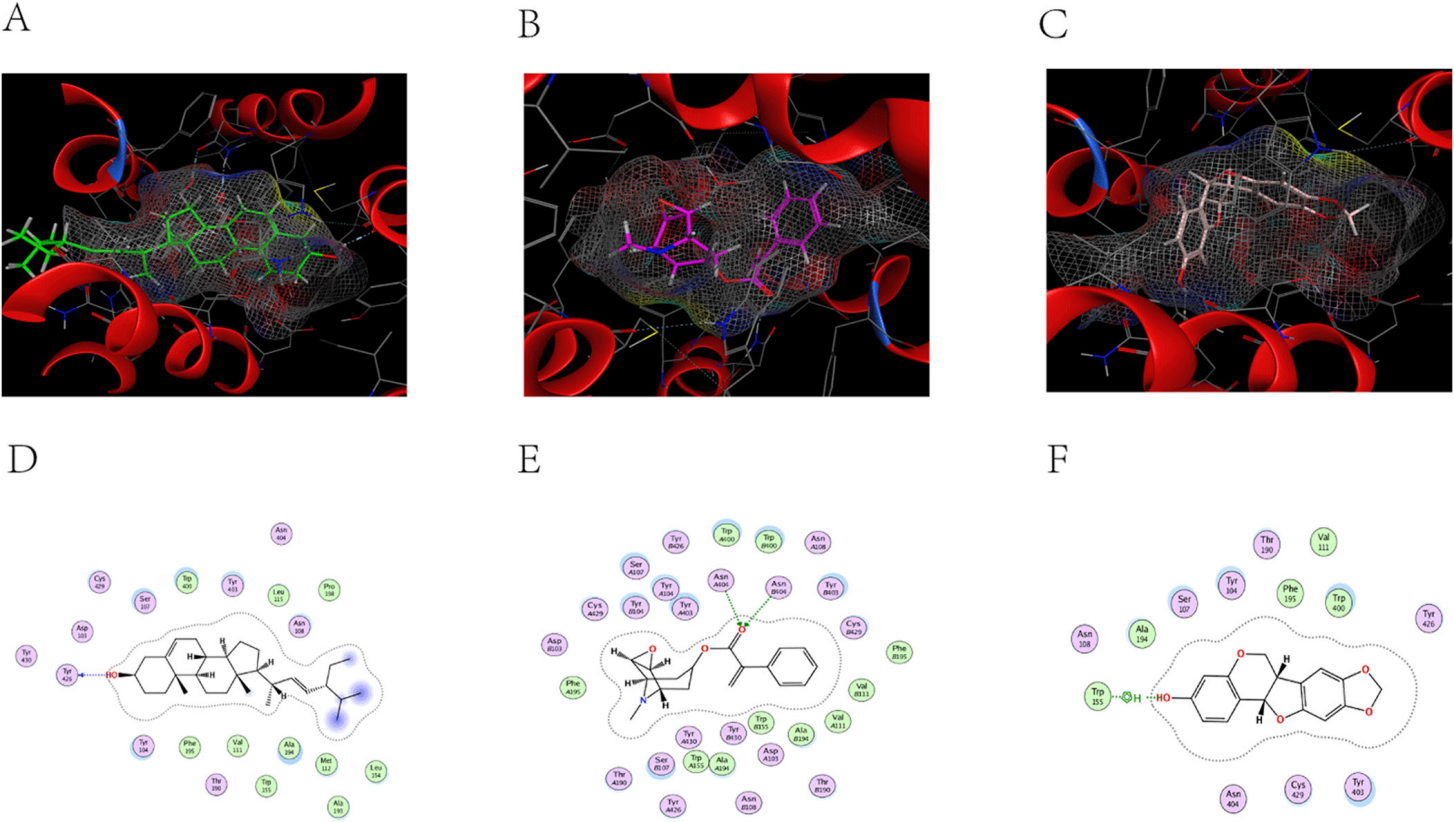
The docking model of compounds with CHRM2.

The structure of CHRM2 was a combination of stigmasterol, aposcopolamine and inermin (Figure 10). Stigmasterol TYR 426 was linked to CHRM2 by hydrogen bonds (Figure 10(D)). Similarly, ASN 404 (A) and ASN 404 (B) of aposcopolamine were linked to CHRM2 by hydrogen bonds (Figure 9(E)). Inermin TRP 155 and CHRM2 were connected by π-H bonds (Figure 10(F)).

Molecular docking was found to mimic the binding ability of different bound compounds and proteins. Table 3 shows the energy docking scores. These results indicated that several KXS compounds can bind well to target genes.

**Table 3.** KXS molecular docking energy scoring results (kcal/mol).

_Receptors_^Ligands^	Original Ligands	Stigmasterol	Aposcopolamine	Inermin
ACHE	−5.3007	−5.1831	−4.4377	−4.4852
ADRA2A	−11.8074	−10.5416	−7.4629	−7.1188
CHRM2	−8.1719	−1.7511	−16.8981	−6.3309

## Discussion

To the best of our knowledge, this is the first study to integrate meta-analysis, network pharmacology and molecular docking to investigate the efficacy and potential pharmacological mechanisms of Kai-Xin-San on AD treatment. The meta-analysis results revealed that KXS could significantly reduce the time of escape latency as well as increase the number of cross-platform and proportion of time in the target quadrant in AD animal models. The network pharmacological analysis of KXS identified 4 herbs, 25 compounds, and 44 target gene-regulated major pathways related to AD. Specific, key genes (DRD2, MAOA, ACHE, ADRA2A) were found to regulate the neuroactive ligand-receptor as well as other numerous signalling pathways. Moreover, molecular docking results indicated that stigmasterol, aposcopolamine and inermin exhibited good affinity for ADRA2A, CHRM2 and ACHE, respectively.

This is the first meta-analysis that thoroughly collated existing data and measured the current preclinical evidence supporting the efficacy of KXS in providing meaningful cognitive benefits in AD treatment. Despite the presence of small-study effects and statistical heterogeneity among studies, our findings demonstrated that KXS exerts a protective effect on retention memory and acquisition memory in experimental AD. This provides a baseline for future research related to KXS-based therapy in AD treatment.

Among the identified compounds, terpenoids and alkaloids play an important role in treatment of AD. Stigmasterol is a terpenoid that reduce production of Aβ thereby protecting the nerves *in vivo* (Burg et al. 2013). Moreover, it has been reported to down-regulate inducible nitric oxide synthase and phosphorylation of factor-κB as well as exert anti-apoptotic and anti-inflammatory effects (Lee et al. 2018). In the same line, fumarine has been reported to inhibit acetylcholinesterase activity and reduce memory impairment (Kim et al. 1999). Furthermore, ginsenoside-rh4 was found to be involved in anti-inflammatory, antioxidant, and neuronal protection (Razgonova et al. 2019; Wang et al. 2019). Collectively, these findings suggested that the main KXS components are effective for treating AD.

The core genes in PPI networks were DRD2, MAOA, ACHE, ADRA2A and CHRM2. Clinical studies have shown that changes in expression of DRD2 affect regional brain capacity thus increasing the risk of developing AD (Roussotte et al. 2015; Blum et al. 2018). MAOA plays a vital role in the neuronal activity of the central nervous system and peripheral tissues as well as in metabolism of vasoactive amines (Naoi et al. 2016). In the same line, ACHE is involved in normal nerve signal transmission thus promoting neuronal development and nerve regeneration (Soreq and Seidman 2001). Adrenergic receptors (ADRA2A) and muscarinic acetylcholine receptors (CHRM2) are members of the G-protein coupled receptor superfamily. They are associated with memory and cognition (Thal et al. 2016).

Molecular docking reveals the interaction between components and targets in a network, thereby improve the accuracy of the network (He et al. 2019). Compared with the original ligand, the binding ability of aposcopolamine was significantly higher than that of its ligand, and the binding ability of stigmasterol and iinermin was slightly lower than that of its ligand. The negative score of the bonds’ binding energy was used to interpret the docking results. In ACHE–stigmasterol interaction, the binding energy of the hydrogen bond and the residue Gly 342 was −5.1831 kcal/mol. This indicated a more stable structure. Stigmasterol was connected to ADRA2A through π-H bonds at C11 at a distance of 4.53 Å and a combined energy of −10.5416 kcal/mol. This connection was stronger compared to that of aposcopolamine-ADRA2A and inermin-ADRA2A. The combined energy of aposcopolamine-CHRM2 was −16.8981 kcal/mol while that of stigmasterol-CHRM2 was −1.7511 kcal/mol. These results strongly suggested that active KXS compounds can effectively treat AD by binding core genes.

KEGG analysis revealed that KXS exerted a therapeutic effect on AD mainly by neuroactive ligand-receptor interaction, regulation of cholinergic synapses, the cGMP/PKG pathway and calcium signalling. Cholinergic transmission is critically important for memory, learning and attention (Hampel et al. 2018). For example, M1 muscarinic agonists decrease Aβ-induced apoptosis and tau hyperphosphorylation (Fisher 2012). In the same line, M1, M3, and M5 inhibit γ-aminobutyric acid-ergic and stimulate glutamatergic neurotransmission (Ferreira-Vieira et al. 2016). The approval of a cholinesterase inhibitor by the Food and Drug Administration (USA) for AD treatment demonstrates the importance of the cholinergic transmission systems (Fisher 2012). Activation of the cGMP/PKG pathway induces the expression of thioredoxin and Bcl-2, which antagonises Aβ toxicity thereby providing neuroprotection (Ju et al. 2005). It also enhances synaptic transmission and brain-derived neurotrophic factor transcription (Sierksma et al. 2013). Calcium ion disorders lead to synaptic loss (Jha et al. 2017). They are closely associated with Aβ aggregation, tau phosphorylation, oxidative stress and inflammation (Alzheimer's Association Calcium Hypothesis 2017).

Nevertheless, further pharmacological experiments are needed to validate the therapeutic mechanism of KXS because this study was based on data analysis only.

## Conclusions

Herein, meta-analysis revealed that KXS improves the cognitive benefits in AD models by reducing the time of escape latency as well as increasing the number of cross-platform and proportion of time in the target quadrant. Network pharmacology identified 25 KXS compounds and 44 genes targeted by KXS. The main bioactive compounds of KXS were: stigmasterol, aposcopolamine, fumarine, and inermin. In the same line, the core genes targeted by KXS compounds were: DRD2, MAOA, ACHE, ADRA2A and CHRM2. KXS was found to be involved in AD treatment by regulating cholinergic synapses, the cGMP/PKG pathway and calcium signalling. Molecular docking results further revealed that several KXS compounds exhibited a good affinity for their core targets. Evidently, KXS exerts a therapeutic effect on AD through a multi-target, multi-component and multi-pathway mechanism.
